# Students’ motivation, study-effort and perceptions of teachers’ goals when engaging in a learning design within the flipped classroom

**DOI:** 10.1186/s12909-025-07729-z

**Published:** 2025-08-12

**Authors:** Hanne Kristine Skjesol Torbergsen, Tove Engan Paulsby, Gørill Haugan, Britt Karin Utvær

**Affiliations:** 1 Department of Public Health and Nursing, NTNU Norwegian University of Science and Technology, Post office box 8905, Trondheim, N-7491 Norway; 2 Faculty of Nursing and Health Science, Nord University, Post office box, Bodø, 1490, 8049 Norway

**Keywords:** Learning design, Flipped classroom, Nursing students, Motivation, Perception of teachers’ goals, Study-effort, Perceived learning outcomes, Achievement goal theory, Self-determination theory

## Abstract

**Background:**

A bachelor’s degree in nursing at a large university in Norway has designed and developed a specific learning design within a flipped classroom to engage and motivate nursing students in their learning of cardiopulmonary resuscitation (CPR), facilitating progression throughout the three academic years of the nursing program. This comprehensive learning design is perceived as a cohesive unit and consists of two pre-class activities and two in-class activities. The four learning activities are multiple choice questions (MCQ), skills training with Resuscitation Quality Improvement (RQI), team-based learning (TBL) and simulation.

**Aims:**

To investigate the associations between nursing students’ autonomous motivation when performing four learning activities in a flipped classroom design and their perceptions of teachers’ goals, study-effort and perceived learning outcomes.

**Methods:**

Quantitative data were tested by means of stepwise regression analysis. Using a cross-sectional design, data were collected from 374 nursing students. Three hypotheses of the associations between nursing students’ autonomous motivation when performing four learning activities in the flipped classroom learning design and their perceptions of teachers’ goals, study-effort and perceived learning outcomes.

**Results:**

There are significant associations between students’ perceptions of teachers’ goals and study-effort and their perceived learning outcomes when they perform the four learning activities. The correlations between students’ autonomous motivation to perform the four learning activities and their perceived learning outcomes in CPR are positive and significant. The regression model explained 32% of the variance in perceived learning outcomes, with students’ perceptions of teachers’ goals and study-effort showing the strongest associations (β = 27 and β = 21, respectively).

**Conclusion:**

This study provides empirical knowledge of the impact of students’ autonomous motivation when performing four learning activities and their perceived learning outcomes. Based on the results of this study, the use of a learning design containing pre-class and in-class activities seems to support nursing students’ learning processes.

**Supplementary Information:**

The online version contains supplementary material available at 10.1186/s12909-025-07729-z.

## Background

### Introduction

Competence in cardiopulmonary resuscitation (CPR) is a critical requirement for both registered nurses and nursing students. Mastery of CPR demands not only up-to-date theoretical knowledge and technical skills but also confidence and the ability to collaborate effectively in interprofessional teams [1]. To support the development of these essential competencies, the Norwegian University of Science and Technology (NTNU) has implemented a learning approach inspired by the flipped classroom model in its bachelor’s degree program in nursing [2, 3]. It includes two pre-class and two in-class activities, aiming to enhance student motivation, engagement, and competence in CPR. This design not only integrates theory and practice but also promotes continuous learning and real-world readiness.

This article addresses a pressing need in nursing education by presenting a structured and sustainable learning design based on the flipped classroom approach. The CPR course described herein serves as a concrete example of this model. While previous research has identified the limitations of traditional teaching methods [1–4], few studies have proposed comprehensive educational frameworks that effectively integrate theoretical instruction with practical application.

By introducing a flipped classroom model tailored specifically to CPR training this study contributes to the growing body of literature on active learning in healthcare. The model offers a replicable framework that enhances knowledge retention, skill acquisition, and clinical preparedness among nursing students. Its emphasis on student motivation, engagement, and real-world relevance represents a meaningful advancement in educational design, with potential implications for curriculum development and patient safety.

### The flipped classroom approach

In the education of nurses, there has been a transition from traditional classroom teaching to the flipped classroom approach [1, 5]. This pedagogical approach is characterized by pre-class preparation, in-class engagement, student-centered and interactive learning, and flexible learning pathways. Within this model, the teacher’s role transitions from being the primary source of knowledge to that of a facilitator and guide. Overall, the flipped classroom is designed to foster deeper understanding, critical thinking [4], as well as improving engagement, student performance and learning outcomes [4, 6, 7].

Building on the benefits of the flipped classroom approach, studies have shown that it positively influences nursing students’ motivation and learning [5, 7–11]. However, a successful implementation of the flipped classroom requires a structured design which will help students identify their learning objectives and understand how to achieve them [7, 12]. At the same time studies have also reported challenges associated with the approach—particularly during pre-class activities. These include increased study-effort, unfamiliarity with the format, and a lack of motivation among students [3, 13].

In the context of CPR education, various multimodal strategies have been explored to improve training outcomes. These include the use of real-time feedback technologies, self-instructional materials, online modules, and interactive simulations [14–17]. Despite these innovations, research consistently shows that CPR competence among nursing students declines rapidly after initial instruction. Even after completing their CPR training, nursing students often struggle to apply their skills effectively [17, 18].

A meta-analysis by Zeng et al. [19] found that high-fidelity simulation improved knowledge and skill performance compared to low-fidelity simulation and traditional manikin-based training. However, the benefits of high-fidelity simulation diminished over time, with no significant advantage in knowledge retention after three months or in skill performance after one year [19].

To address these challenges, the flipped classroom offers distinct advantages for CPR instruction. By combining theoretical preparation with repeated hands-on practice, this approach supports long-term retention and progressive skill development. At NTNU, the structured learning design was implemented across all three years of the nursing program aiming to integrate theory and practice as well as promoting continuous learning and real-world readiness.

### The flipped classroom learning design

There was a clear need to revise the traditional approach to a flipped classroom model due to several persistent challenges. Conventional methods, which often rely on lectures and isolated skills training sessions, have shown limited effectiveness in promoting long-term retention of both theoretical knowledge and psychomotor skills. Research indicates that students tend to forget critical CPR competencies shortly after training [17, 18], posing a significant risk in clinical practice where rapid and accurate responses are essential.

Furthermore, traditional teaching formats often fail to actively engage students or connect learning to real-life clinical scenarios, resulting in fragmented understanding and limited transferability of knowledge [17, 18]. To address these challenges, we developed a more integrated, student-centered learning model grounded in the course’s defined learning outcomes. These outcomes encompass theoretical knowledge, practical skills, and general competence, outlining the level of proficiency students are expected to achieve by the end of the course. This revised approach not only facilitates knowledge acquisition but also cultivates critical thinking, decision-making, and teamwork—competencies essential for effective performance in cardiac arrest situations. The learning design consists of four interrelated activities—two pre-class and two in-class—deliberately structured to form a cohesive and progressive learning experience. This structure ensures that students build competence incrementally, with each activity reinforcing and expanding upon the previous one. The design is implemented consistently across all three years of the nursing program to support continuous learning and prevent the decline of CPR knowledge and skills over time [17, 18].

It combines non-mandatory pre-class activities with mandatory in-class activities, aiming to enhance students’ competence in assessments, decision-making, and actions related to CPR. This design begins with a theoretical individual study activity including recommended course material and culminates in simulation-based practicums where students work in teams, mimicking real-world nursing environments.

The four activities are:

1. Multiple-choice questions (MCQ): Based on European Resuscitation Council (ERC) guidelines, these questions are made available on the learning platform four weeks prior to the in-class sessions. Students are encouraged to use course literature and can complete the quiz multiple times within a set timeframe. Although optional, most students complete it once, typically in about one hour.
2. Skills training with Resuscitation Quality Improvement (RQI). This skills training involves a mannequin-based station that provides real-time, computer-generated feedback on chest compressions and ventilations. The RQI module is available three weeks before the in-class sessions and must be completed beforehand. Students usually spend around 30 min on this activity.
3. Team-based learning (TBL). Facilitated by instructors, this session involves collaborative problem-solving in small groups. Students apply their theoretical knowledge to case-based discussions aligned with the learning outcomes of the CPR course. The session is scheduled and lasts approximately 2.5 h.
4. Simulation (low- to high-fidelity). Conducted in realistic clinical settings, this activity requires students to work in teams to manage simulated cardiac arrest scenarios. It integrates knowledge from the MCQ and TBL sessions with hands-on skills from the RQI training. This session is also scheduled and lasts 2.5 h.

The design evolves through the three years of study. In the first year, the focus is on basic CPR skills. In the second and third years, the emphasis shifts to communication, teamwork in acute care settings, and advanced CPR techniques. The pre-class activities (MCQ and RQI) are non-mandatory but strongly encouraged, while the in-class activities (TBL and simulation) are mandatory and scheduled within a two- to six-week window.

This structured and longitudinal approach aims to foster deeper engagement, promote autonomy, and ensure that students are well-prepared to respond effectively in real-world cardiac emergencies.

### Theoretical frameworks

#### Achievement goal theory and self-determination theory

When studying student engagement and motivation, both achievement goal theory and self-determination theory are central theoretical frameworks [20, 21]. These frameworks emphasize the importance of providing students with appropriately challenging academic tasks and fostering a supportive learning environment where teachers act as facilitators rather than mere transmitters of knowledge [20]. Research shows that students are more likely to be motivated when they perceive their teachers as supportive of their learning goals [22–24]. These theories offer complementary perspectives on how motivation is shaped by both individual dispositions and contextual factors.

#### Achievement goal theory

Achievement goal theory as described in Anderman et al. [25], explains why and how students engage in academic activities and their perceptions of contexts that affect their engagement and motivation. The theory distinguishes between two primary goal orientations: mastery goals and performance goals, each with approaching and avoidance components [25, 26].

Mastery goals refer to students’ eagerness for understanding and personal improvement, whereas performance goals refer to the desire to outperform others [25]. The personal dimension relates to students’ ability to achieve competence in learning situations [27]. The contextual aspect relates to classroom goal structures. This represents the teacher’s goal-related communication and the student’s perception of their teachers’ goals [20, 24, 26, 28].

Existing evidence indicates that a classroom mastery goal structure positively influences students’ own mastery goals [29]. Students construct meaning for performing learning activities based on their classroom perceptions [28]. Learning outcomes, fellow students, and teachers’ pedagogical approaches affect students’ quality of engagement in learning activities [28]. Teachers who are mastery-focused promote a positive interpersonal climate, engaging in motivationally supportive interactions with students [26]. Such interactions include encouraging and supporting students, being persistent, using humor, and showing enthusiasm. Thus, the main goal is to develop competence, which is associated with motivation, positive emotions, and positive student-teacher relationships [21, 26].

Students oriented towards mastery goals are more likely to engage in behaviours that promote achievement [26, 28]. The perception of success varies among students. When success is defined as learning, understanding, and skill development, students engage more deeply in classroom activities and demonstrate resilience in the face of learning challenges. Conversely, if success is seen as outperforming others, students may resort to shortcuts to avoid difficult tasks, leading to superficial engagement and learning [21, 26].

#### Self- determination theory

Self-Determination Theory (SDT), developed by Ryan and Deci [30], provides a comprehensive framework for understanding human motivation. It emphasizes the quality of motivation, distinguishing between autonomous and controlled forms along a continuum of internalization [30–32].

According to SDT, students have three basic psychological needs: autonomy, perceived competence and relatedness [23, 30]. The fulfillment of all three needs is essential for fostering effective learning and promoting optimal personal development [23, 33]. Autonomy is supported when students are offered meaningful choices within a structured learning environment—for instance, by allowing them to determine how they approach their learning activities. A sense of competence arises when students overcome academic challenges, understand the course material, and perceive progress in their skills [23, 31].

Furthermore, SDT emphasizes that behavior is shaped within a social context [23]. When students experience a sense of relatedness—that is, when they feel connected to peers or teachers—they are more likely to engage deeply with the learning process and experience enhanced motivation and growth [23, 33].

Autonomous motivation is defined as engaging in activities for inherent interest and enjoyment [23, 30]. It includes intrinsic motivation as well as integrated and identified regulation. In contrast, controlled motivation arises from pressure and demands. The more internalized the motivation is, the more it becomes part of a student’s identity. When motivation is internalized, students are more likely to see their learning activities as aligned with their personal values and interests, leading to deeper engagement and sustained effort. Thus, autonomous motivation is also associated with higher study-effort, and consequently better learning [23, 30].

SDT thus provides a broad framework for understanding aspects that facilitate or undermine autonomous motivation, and learning, which are directly relevant to educational settings. Research shows that both intrinsic motivation and well-internalized forms of extrinsic motivation predict positive outcomes across educational levels and cultural contexts [30].

#### Linking the frameworks

Several studies have demonstrated a strong association between autonomous motivation and mastery goals [34–36]. Chen et al. [35] reported that the mastery goals approach was related to intrinsic motivation among university students, and that achievement goals mediated the need for support. A study by Rothes et al. [36] showed that both mastery goals and autonomous motivation were significant predictors of student engagement and learning [36].

Together, these frameworks provide a robust foundation for examining how flipped classroom designs can enhance motivation, engagement, and learning outcomes in nursing education.

#### Present study

This study investigates nursing students undergoing CPR training within a structured flipped classroom learning design. The design is intentionally aligned with principles of autonomous motivation and mastery goal orientation, aiming to enhance both theoretical knowledge and practical skills in CPR.

By examining how students’ motivation, study-effort, and perceptions of teachers’ goals relate to their perceived learning outcomes, this research contributes to a deeper understanding of how flipped classroom models can support competence development in nursing education. The study offers empirical insights into the mechanisms that underpin effective learning in practice-oriented settings and provides evidence to inform the development of innovative, student-centered teaching strategies.

#### The research question explored is

How do students’ motivation, study-effort and perceptions of teachers’ goals associate with their perceived learning outcomes in a flipped classroom learning design?

#### Contribution to the field

By exploring these dimensions, the study aims to advance the pedagogical foundations of nursing education and support the implementation of evidence-based teaching practices that promote lifelong learning and clinical competence.

This study is significant for several reasons:

1. It addresses a critical educational need: Improving CPR competence among nursing students is essential, as these skills are directly linked to patient survival in emergency care.
2. It explores the role of motivation and instructional alignment: Understanding how students’ internal motivation and their perceptions of teacher support influence learning outcomes can inform more effective teaching practices.
3. It provides a model for evidence-based curriculum development: The findings may guide the implementation of active learning strategies within flipped classroom designs that foster lifelong learning and clinical readiness.

#### Aims and hypotheses

The aim of this study was to investigate the associations between nursing students’ autonomous motivation when performing the four learning activities in the flipped classroom learning design and their perceptions of teachers’ goals, study-effort and perceived learning outcomes. By using CPR as an educational case, the following three hypotheses were tested via stepwise regression analysis.

1. When students perform four learning activities, autonomous motivation affects their perceived learning outcomes (PLO).
2. Perceptions of teachers’ goals (PTGs) affect students’ perceived learning outcomes.
3. Study-effort affects students’ perceived learning outcomes.

**Fig. 1:**
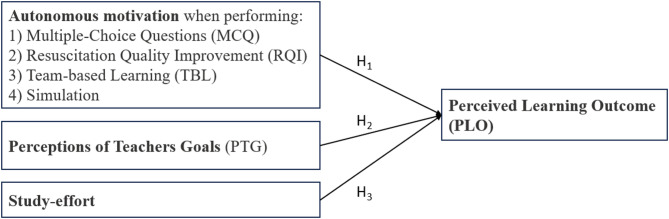
The tested hypothesized associations

## Methods

### Participants and procedure

During autumn 2019 and spring 2020, cross-sectional data were collected via a questionnaire at a large university in Norway. All bachelor-level nursing students across the three years of study were invited to participate. A pilot study conducted in 2018 helped refine the questionnaire by reducing redundancy and improving clarity based on feedback from students and faculty.

Due to the COVID-19 pandemic, data collection transitioned from in-person classroom distribution to online forms, which likely impacted the response rate—particularly among second-year students, as the survey coincided with the national lockdown in March 2020.

Ethical approval was obtained from SIKT—The Norwegian Agency for Shared Services in Education and Research—and the university’s management unit. The study adhered to the principles outlined in the Declaration of Helsinki and complied with Norwegian data protection laws. Participation was voluntary, anonymous, and uncompensated. Informed consent was implied through completion of the survey, which was promoted via classroom announcements, email, and the university’s learning platform (Blackboard).

We have chosen to describe the survey items and some supplementary analysis through three additional files. The additional files 1 and 2 show all items used in our analyses whereas the additional file 3 demonstrates the explorative factor analysis for the involved measurements scales.

### Measurements

The following measurement scales were included in the presented order.

#### Autonomous motivation when performing specific learning activities

Autonomous motivation was assessed by three items theoretically rooted in Self-determination theory [23], including the characteristics of intrinsic, integrated and identified regulation. These items were developed for this study by the researchers involved. Intrinsic regulation pertains to activities done “for their own sake” or for their inherent interest and enjoyment. The identified regulations reflect that the person consciously identifies with, or personally endorses, the value of an activity and thus experiences a relatively high degree of volition or willingness to act, whereas integrated regulation reflects that the person, in addition to recognition and identification with the value of the activity, also finds it to be congruent with other core interests and values. Integrated and identified regulations are well internalized and thus autonomous forms of extrinsic motivation—people view activities as worthwhile even if not enjoyable [23, 37]. On a five-point scale ranging from 1 (very little) to 5 (very much), the present study assessed autonomous motivation with three items: [1] fun/enjoyable [2], useful and [3] important.

#### Perception of teachers’ goals

The patterns of adaptive learning scales were designed by Midgley and colleagues [38] and include several scales, including both mastery and performance goals, as well as the separation between performance-approach and performance-avoidance dimensions. In this study, only items of the teacher mastery goal are included, referring to the student’s perception of the goal, as contextual cues from their teachers. These five items range on a seven-point scale from 1 (strongly disagree) to 7 (strongly agree). Examples of sample items are “*The teachers think mistakes are okay as long as we are learning”* and “*the teachers recognize us for trying hard”*. Teacher mastery goal has previously reported a Chronbach’s alpha value of 0.83 [38].

#### Study-effort

The Intrinsic Motivation Inventory [39, 40] includes a subscale with three items assessing study-effort on a seven-point scale ranging from 1 (strongly disagree) to 7 (strongly agree). This variable is important when investigating motivation in specific issues and contexts. The measurement scale of study-effort assesses the person’s investment of his/her capacities in what he/she is doing. This subscale was previously validated among college students reporting Alpha 0.78 [41]. Examples of items are “*I tried very hard to do well in this course”* and *“I put a lot of effort into this course”*. One additional item, constructed for this specific learning design, was also included: “*I actively took part in the discussions and reflection in this course*.”. This is because both team-based learning and simulation involve discussions and reflections with the teachers and peers.

### Students’ perceived learning outcomes

Eleven items were designed for this study to determine the degree to which the students perceived that they had reached the defined learning outcomes of the CPR course. These items were stated as learning outcomes in the curriculum for the CRP course. The items were assessed on a five-point scale ranging from 1 (very little) to 5 (very much). Example items include *“By attending this course*,* my knowledge regarding situations in relation to cardiac arrest has improved”*, *“By attending this course*,* my practical skills in CPR have improved”*, and *“By attending this course*,* my competence in working in stressful contexts has improved”*. Additional files 1 and 2 show the wordings of the items included in this study along with the means, standard deviations, skewness, and kurtosis.

#### Statistical analysis

Descriptive analysis, correlations, and simultaneous regression analysis were conducted via IBS SPSS Statistics version 28. All the factors were introduced via stepwise linear regression to determine which variables significantly predicted the perceived learning outcomes during CPR. All the correlations and regression analyses were conducted with the different concepts mean score.

## Results

### Descriptives

In total, 63% (374 out of 590 students) participated. Those who participated in only one or two of the four learning activities were excluded from the study, resulting in an effective sample of 351 students. Missing data in the other three measurement scales (perceptions of teachers’ goals, study-effort and students’ perceived learning outcomes were replaced by mean scores. Table 1 shows the participant characteristics; more than half (190 = 54%) were 1 st year students, 48 (14%) were 2nd year students, and 113 (32%) were in the third year of the study program. Moreover, 88% (*N* = 307) were women, and 12% (*N* = 44) were men.

**Table 1:** Sample characteristics

	Number of participants (*N*)		Percent total (%)
First study-year	190 (of total 229 students)		54
Second study-year	48 (of total 216 students)		14
Third study-year	113 (of total 145 students)		32
Female	307		88
Male	44		12

*N* = 351

#### Correlations using Pearson’s r

The correlations between students’ autonomous motivation to perform the four learning activities and their perceived learning outcomes in CPR are positive and significant (*p* ≤ 0.01) (Table 2). The simulation showed the strongest association (*r* = 0.38), whereas multiple-choice questions and team-based learning demonstrated somewhat lower correlations, with *r* = 0.31 and 0.30, respectively. The RQI disclosed an *r* = 0.18 with students’ perceived learning outcomes.

Students’ autonomous motivation when performing each of the four learning activities is also significantly correlated with perceptions of teachers’ goals and study-effort. Simulation has the strongest association with perceptions of teachers’ goals (*r* = 0.44), whereas autonomous motivation to perform team-based learning has the strongest correlation with study-effort (*r* = 0.33). In terms of the correlations between all the study variables, perceptions of teachers’ goals and study-effort demonstrated the strongest associations with students’ perceived learning outcomes (*r* = 0.41 and 0.44, respectively). Moreover, the internal consistency was good, with Cronbach’s alpha coefficients ranging between α = 0.70 and 0.89.

**Table 2:** Cronbach's alpha (α) and correlation matrix for the study variables

	MCQ	RQI	TBL	Simulation	PTG	Study-effort	PLO
**MCQ**	1	0.16^**^	0.29^**^	0.19^**^	0.35^**^	0.26^**^	0.31^**^
**RQI**		1	0.15^**^	0.29^**^	0.16^**^	0.24^**^	0.18^**^
**TBL**			1	0.31^**^	0.28^**^	0.33^**^	0.30^**^
**Simulation**				1	0.44^**^	0.30^**^	0.38^**^
**PTG**					1	0.30^**^	0.44^**^
**Study-effort**						1	0.41^**^
**PLO**							1
**Cronbach's alpha (α)**	0.84	0.80	0.89	0.86	0.78	0.70	0.89
**Items**	3	3	3	3	5	4	11

*p ≤ 0.05, ** *p* ≤ 0.01. *MCQ* Multiple-Choice Questions, *RQI* Resuscitation Quality Improvement, *TBL* Team-based Learning, *PTG* Perceptions of Teacher's Goals, and *PLO* Perceived Learning Outcomes. *N* = 351

#### Stepwise multiple regression: associations with students’ perceived learning outcomes

To investigate which aspects are most influential on students’ perceived learning outcomes, a stepwise regression model was employed (Table 3). The learning activities are included in the model in the same order as in the learning design. In step 1, students’ autonomous motivation when performing multiple-choice questions was included. Multiple-choice questions accounted for 9% of the variance in students’ perceived learning outcomes and remained significant throughout the entire regression analysis. These findings indicate a clear association with students’ perceived learning outcomes. When step 2 is entered, the RQI shows a significant estimate that adds 2% to the explained variance. However, the impact of the RQI decreased as subsequent variables were included. The same pattern was also observed with team-based learning, which was added in step 3. However, when simulation is included as the fourth learning activity (step 4), the model reveals a clear association with perceived learning outcomes. Overall, the four learning activities explained 21% of the variation in the students’ perceived learning outcomes in response to the CPR.

**Table 3:** Stepwise multiple regression: factors associated with students’ perceived learning outcome

		B	SD	beta	t	*p*	Adjusted *R*^2^
Step 1	Constant	2.95	0.10		28.95	<,001	
	MCQ	0.18	0.03	0.31	6.03	<,001	0.09
Step 2	Constant	2.48	0.21		11.88	<,001	
	MCQ	0.17	0.03	0.29	5.59	<,001	
	RQI	12	0.05	0.13	2.59	0.010	0.11
Step 3	Constant	2.27	0.21		10.81	< 0.001	
	MCQ	0.13	0.03	0.23	4.35	< 0.001	
	RQI	0.10	0.04	0.11	2.16	0.032	
	TBL	0.12	0.03	0.22	4.14	< 0.001	0.15
Step 4	Constant	1.89	0.21		8.81	< 0.001	
	MCQ	0.12	0.03	0.20	4.03	< 0.001	
	RQI	0.04	0.04	0.04	0.86	0.391	
	TBL	0.08	0.03	0.15	2.84	0.005	
	Simulering	0.19	0.04	0.28	5.45	< 0.001	0.21
Step 5	Constant	1,48	0.22		6.67	< 0.001	
	MCQ	0.08	0.03	0.13	2.64	0.009	
	RQI	0.04	0.04	0.04	0.90	0.368	
	TBL	0.07	0.03	0.12	2.42	0.016	
	Simulering	0.13	0.04	0.18	3.42	< 0.001	
	PTG	0.17	0.03	0.28	5.13	< 0.001	0.27
Step 6	Constant	1.23	0.22		5.56	< 0.001	
	MCQ	0.06	0.03	0.107	2.18	0.030	
	RQI	0.01	0.04	0.01	0.25	0.803	
	TBL	0.04	0.03	0.07	1.46	0.144	
	Simulering	0.11	0.04	0.16	2.99	0.003	
	PTG	0.15	0.03	0.25	4.68	< 0.001	
	Study-effort	0.14	0.03	0.23	4.61	< 0.001	0.31
Step 7	Constant	0.88	0.27		3.33	< 0.001	
	MCQ	0.08	0.03	0.14	2.72	0.007	
	RQI	0.04	0.04	0.05	1.03	0.303	
	TBL	0.03	0.03	0.05	1.01	0.314	
	Simulering	0.11	0.04	0.15	2.94	0.003	
	PTG	0.17	0.03	0.27	5.17	< 0.001	
	study effort ^5^/_6_	0.13	0.03	0.21	4.20	< 0.001	
	Gender _(1=male, 2=female)_	0.02	0.07	0.01	0.22	0.829	
	Study-year _(1=1st, 3=3rd)_	0.08	0.03	0.14	2.89	0.004	0.32

*p ≤ 0.05, ** *p* ≤ 0.01. *MCQ* Multiple-Choice Questions, *RQI* Resuscitation Quality Improvement, *TBL* Team-based Learning, *PTG* Perceptions of Teacher`s Goals. *N* = 351

Step 5 introduces perceptions of teachers’ goals, contributing a notable 6% increase to the explained variance, and its significance persists through the final steps of the model. Step 6 encompassed the inclusion of study-effort, contributing an additional 4% to the variance. In the final step, sex and study year were included as control variables. The analysis revealed that study year had a significant association with perceived learning outcomes when accounting for all other variables, whereas gender did not exhibit such a relationship. In sum, the regression model explains 32% of the variation in students’ perceived learning outcomes. The factors that are significantly related to perceived learning outcomes throughout the model are the pre-class activity multiple-choice questions, the in-class activity simulation, perceptions of teachers’ goals, the study-effort, and the students’ year of study.

## Discussion

Facilitating students’ motivation and make teachers’ goals clear are important when creating a learning design. It is also fundamental to obtain knowledge on how nursing students learn when they are attending different learning activities within a learning design and how the different learning activities operate together and supplement each other. This specific learning design is perceived as a whole, and the combination of the four learning activities together with teachers supporting students’ mastery goals, intended to inspire to study-effort and thereby enhancing nursing students’ learning and competence development. The learning design encourages students to build knowledge and skills step-by-step and subsequently motivate them to gain theoretical and practical competence in cardiac arrest situations. The learning design starts with a theoretical self-study activity and ends with a simulation-based practicum, aiming to imitate an environment of the real world of nursing.

Several studies have shown that using a flipped classroom approach positively affects students’ motivation and learning [5, 8, 11, 42, 43]. Additionally, evidence indicates that students become motivated to learn when teachers support their learning processes [23, 24, 44].

However, this study provides empirical knowledge about (I) the impact of students’ autonomous motivation when performing four learning activities on their perceived learning outcomes; (II) the empirical associations between students’ perceptions of teachers’ goals and their perceived learning outcomes when performing the four learning activities; and (III) the associations between the four learning activities within the flipped classroom approach and nursing students’ autonomous motivation, their study-effort and their perceived learning outcomes. The results gave support to all three hypotheses even through some of the coefficients were quite weak.

### Students’ autonomous motivation when performing the learning activities

The specific learning design consists of two pre-class activities and two in-class activities and is to be considered in combination. The correlations between students’ autonomous motivation when performing the four learning activities and their perceived learning outcomes in CPR are positive and significant. Simulation revealed the strongest correlation (*r* = 0.38**) followed by multiple choice questions and team- based learning (*r* = 0.31 and *r* = 0.30 respectively). It is not surprising that simulation, which is the fourth and last learning activity in the design, is the activity students perceive as having the strongest correlation with their learning outcomes in the CPR course. Several studies support that simulation, with its hand-on approach, enhances motivation and learning [45]. In addition, since simulation was the final activity, implementing knowledge and skills from the three previous ones, simulation will probably gain some added value, which is visible in the regression model.

In the stepwise multiple regression analysis, the explained variance increases with each of the four learning activities. When adding additional variables, their added value to the model will depend on which number in the series the variables are added. Overall, the combination of the four learning activities explained 21% of the variance in the students’ perceived learning outcomes (step 4), indicating that these activities play a significant role in shaping students’ learning experiences. However, an explained variance of 21% also indicates that several other factors contribute to their perceived learning outcomes in this course, something we will return to later.

Multiple-choice questions, skills training with RQI, and team-based learning help students in simulations apply and develop knowledge and skills in cardiac arrest scenarios. In simulation, students apply the competence they have acquired by participating in previous learning activities. Multiple-choice questions and skills training give students choices when conducting preparatory pre-class learning activities.

To contribute to the experience of autonomy, students can carry out multiple-choice questions and skills training with RQI at any time within a given period and as many times as they wish. In addition, the results suggest that multiple-choice questions support students’ autonomy and mastery, as students are encouraged by teachers to use syllabus literature when solving these problems. The goal is for students to develop knowledge and meet in preparation for team-based learning and simulation. Skills training with RQI helps students maintain and develop skills in CPR. Thus, they practiced their skills in time for simulation. However, a low correlation indicates that students do not recognize skills training with RQI as important as the three other learning activities in the learning design.

Team-based learning has a lower correlation with students’ perceived learning outcomes than simulations. This may suggest that students do not perceive autonomous motivation in team-based learning to the same extent as they do in simulation-based CPR training. In team-based learning, students tend to focus more on developing and safeguarding their knowledge of CPR rather than developing their CPR skills. Although team-based learning is considered a useful and fun form of learning, some studies have shown that students experience that much work is required in advance [3, 13], which can influence motivation.

Discussions as part of in-class activity have been reported as positive experiences in some studies [3, 13]. This finding demonstrates that using a flipped classroom approach in both pre-class and in-class activities may help nursing students develop competence beyond just gaining theoretical knowledge. In nursing education, competencies such as collaborative skills in acute and complex situations, working in stressful environments and ethical competence are not achieved by reading and studying books alone. These skills are also attained by training and exercising together with other students as well as teachers [1]. Therefore, this specific learning design has advantages in that it focuses on the development of different skills and offers a variety of learning activities. A study by Torbergsen et al. [46] revealed that supporting first-year nursing students’ autonomy affects their intrinsic motivation and perception of achieved learning outcomes.

However, our results indicate that undertaking these four learning activities might enhance students perceived learning outcomes. By attending a learning design that emphasizes mastering knowledge and skills, students may feel recognized as individuals and not just recipients of teaching and transfer of knowledge. Additionally, there is a progression of knowledge and skills through three years of education, which will enable students to solve challenges effectively.

### Students’ perceptions of teachers’ goals

Evidence has shown that to support students’ autonomy and consequently their autonomous motivation, teachers using the flipped-classroom approach should provide a clear structure to the course [33, 41]. A study among 350 South Korean university students attending twelve different courses via a flipped classroom approach indicated that teacher facilitation, such as leading discussions and providing feedback, also contributed to students’ enjoyment in class [43]. Thus, the teacher’s role and importance are extremely important.

Also, it is important that teachers justify why the specific design has been chosen [47, 48]. This helps students become more aware of what is needed to acquire learning. Research shows that it is important to communicate clear frameworks and requirements to students so that they know what professional competencies are required to become nurses. In a study by Torbergsen et al. [46], the absence of structure and study requirements contributed to lower motivation and study-effort. Teachers who support and facilitate students in their learning activities can significantly enhance their learning. This includes understanding the content and feeling that they master the content well [46].

The measurement items within the concept of perceptions of teachers’ goals have mean scores ranging from 4.44 to 5.69, as shown in the Additional file 2, which demonstrates that the students’ experiences of teachers’ support and facilitation in the four learning activities are quite high. Students’ perceptions of teachers’ goals contribute an additional 6% to the explained variance of students perceived learning outcomes when incorporated into the model (step 5). The results indicate that teachers who emphasize learning over memorization, are associated with higher perceived learning outcomes among students. Likewise, the results are in line with self-determination theory, which highlights the importance of fulfilling students’ need needs of autonomy, competence and relatedness [23]. Furthermore, our results suggest that teachers supporting students’ needs through emphasizing mastery goals can enhance students’ autonomous motivation. This is especially the case when teachers listen to students and provide them with support during learning activities.

### Study-effort and study years when completing the learning activities

Some studies indicate that students find that student-active learning activities are time-consuming and that they require considerable study-effort [13, 49]. These studies reported a greater workload, use of time, lack of meaning and lack of motivation while performing preparatory activities [13]. In our study, when the study-effort was added to the model, the explained variance increased with 4% (step 6). The present study revealed that students experience the four student-active forms of learning as fun, interesting and useful also put more effort into the CRP course and took actively part in the discussions. A survey among medical students revealed that autonomous motivation is associated with greater study-effort [50] and therefore better learning.

Additionally, our study indicates that study year might influence the experience of learning outcomes. Study year and gender added 1% to the models’ explained variance. However, among these two variables, only study year revealed a significant effect (step 7). The higher percentage of students in their first year of nursing education compared to those in their second or third year, might indicate that first-year students align more closely with the learning design. They may be more adaptable and less set in their ways than more experienced students. However, third-year students experience higher learning outcomes compared to first-year students. Subsequently, they understand more and handle challenges better as they progress further in their studies.

#### Strengths andlimitations

This study tested the associations between nursing students’ autonomous motivation when performing the four learning activities in the flipped classroom learning design and their perceptions of teachers’ goals, study-efforts and perceived learning outcomes via correlations and stepwise multiple regression analysis.

A strength of this study is the examination of associations that have not been previously tested among nursing students. In doing so, we are building a strong theoretical foundation to improve practice with measurement scales that possess reliable psychometric properties combined with measurements created to cover the rationale for this specific study (e.g. Perceived learning outcomes based on the learning objectives for the CRP course). Researching one’s own practice to develop effective learning design and promote research-based education is an explicit educational policy objective [51, 52].

However, some limitations must be kept in mind. The study does not account for the possibility that students might exhibit other types of motivation and regulations beyond the autonomous types. For example, controlled motivation or amotivation [23, 32] do not take part of our research design. We chose autonomous motivation for this study because, in our experience, students often value CPR as an important competence related to becoming a nurse. However, there might be cases where controlled motivation types or amotivation are dominant among nursing students. Including more types of students’ motivation could provide additional and valuable insights into other associations that contribute to understanding students’ perceived learning outcomes, but it requires that several more items are included in the questionnaire.

Similarly, performance goals are not included in the questionnaire, we have only focused on mastery goals [21]. The primary reason for this is that we were interested in exploring the associations between mastery goals and autonomous motivation. As pointed out above, we had to be selective due to the scope of items.

As a study with a cross-sectional design, it does not allow conclusions to be drawn about causality. A longitudinal design would have allowed changes to be assessed over time. Although a stepwise regression analysis can be sensitive to the sample size, it analyses only a dependent variable with independent variables. However, the sample size is considered large, capturing some of the complex phenomena in explaining students’ motivation for learning. By including independent variables such as the four learning activities, perceptions of teachers’ goals, study-effort, gender and study year, we can account for factors that influence students’ perceived learning outcomes when they attend different learning activities. Nevertheless, we acknowledge that the explained variance is only 32% and that there are several other factors, not included in the model, also influence students’ perceived learning outcomes. We are also aware that this specific learning design can be one out of more constructive learning design for CRP for bachelor nursing students. This being said, we have explored the flipped classroom approach and found it to be constructive based on several years of experience at NTNU.

## Conclusion

This article addresses a pressing need in nursing education by presenting a structured and sustainable learning design based on the flipped classroom approach. The CPR course described herein serves as a concrete example of this model. Based on the results of this study, the use of a learning design containing pre-class and in-class activities influences and supports nursing students’ learning processes.

This specific learning design is perceived as a cohesive unit, consisting of two pre-class activities and two in-class activities. The study provides empirical knowledge of how students’ autonomous motivation influences students’ perceived learning outcomes when performing these four learning activities. Additionally, there are significant associations between students’ perceptions of teachers’ goals and study-effort, and students’ perceived learning outcomes when they carry out the four learning activities.

This educational model offers a structured and sustainable approach to CPR training in nursing education. By integrating active learning principles and aligning theoretical and practical components, the flipped classroom design addresses key limitations of traditional methods. It supports long-term retention, enhances clinical competence, and prepares students for real-life cardiac arrest situations. Additionally, it provides a replicable framework for other institutions seeking to improve CPR learning and patient safety outcomes.

The pre-class activities emphasize student engagement and motivation, as well as flexibility. Allowing students to prepare at their own pace and according to their individual schedules. Also giving students, the opportunity to come prepared to the in-class activities, thus enabling them to take responsibility for one’s own learning.

By implementing strategies for effectively communicating mastery goals to support students and encourage them to put effort into the course and actively take part in the discussions, nursing educators can create a more engaging and effective learning environments. These learning environments not only motivates students but may also enhance their learning outcomes. However, further research is recommended to explore this relationship.

## Supplementary Information

Supplementary Material 1.

Supplementary Material 2.

Supplementary Material 3.

## Data Availability

The datasets used and/or analysed during the current study are available from the corresponding author on reasonable request.We have chosen to describe the survey through additional files 1, 2 and 3. The additional files show all questions used in our analyses.

## Abbreviations

CPR: cardiopulmonary resuscitation
ERC: European Resuscitation Council
RQI: Resuscitation Quality Improvement
MCQ: multiple choice questions
TBL: team-based learning
PLO: perceived learning outcomes
PTG: perceptions of teachers’ goals
SIKT: The Norwegian Agency for Shared Services in Education and Research
