# Supplementary material for: Students’ motivation, study-effort and perceptions of teachers’ goals when engaging in a learning design within the flipped classroom
Source: BMC Med Educ. 2025 Aug 12;25:1160. doi: 10.1186/s12909-025-07729-z (PMC12341204; doi:10.1186/s12909-025-07729-z)
Supplement: Supplementary file 1 — Supplementary Material 1. [file 12909_2025_7729_MOESM1_ESM.docx]

Additional file 3

Exploratory Factor Analysis of the Measurements

| Concept | Variabel/item | Factor Loadings | Eigenvalues | Percent of the variance |
| --- | --- | --- | --- | --- |
| Autonomous  Motivation (AM) | AM MCQ1 | 0.82 | 2.28 | 76.06 |
|  | AM MCQ2 | 0.92 |  |  |
|  | AM MCQ3 | 0.88 |  |  |
|  | AM RQI1 | 0.81 | 2.20 | 72.43 |
|  | AM RQI2 | 0.90 |  |  |
|  | AM RQI3 | 0.86 |  |  |
|  | AM TBL1 | 0.89 | 2.47 | 82.25 |
|  | AM TBL2 | 0.93 |  |  |
|  | AM TBL3 | 0.92 |  |  |
|  | AM Simulation1 | 0.84 | 2.37 | 78.90 |
|  | AM Simulation1 | 0.92 |  |  |
|  | AM Simulation1 | 0.90 |  |  |
| Percetions of Teachers Goals (PTG) | PTG1 | 0.67 | 2.71 | 54.14 |
|  | PTG2 | 0.74 |  |  |
|  | PTG3 | 0.80 |  |  |
|  | PTG4 | 0.77 |  |  |
|  | PTG5 | 0.68 |  |  |
| Study-effort | Study-effort1 | 0.81 | 2.19 | 54.83 |
|  | Study-effort2 | 0.85 |  |  |
|  | Study-effort3 | 0.72 |  |  |
|  | Study-effort4 | 0.56 |  |  |
| Perceived Learning Outcome  (PLO) | PLO1 | 0.66 | 5.33 | 48.47 |
|  | PLO2 | 0.68 |  |  |
|  | PLO3 | 0.53 |  |  |
|  | PLO4 | 0.70 |  |  |
|  | PLO5 | 0.69 |  |  |
|  | PLO6 | 0.74 |  |  |
|  | PLO7 | 0.77 |  |  |
|  | PLO8 | 0.73 |  |  |
|  | PLO1 | 0.77 |  |  |
|  | PLO10 | 0.73 |  |  |
|  | PLO11 | 0.65 |  |  |

Extraction method: Principal Component Analysis. Rotation: None. N=351
