# Supplementary material for: Students’ motivation, study-effort and perceptions of teachers’ goals when engaging in a learning design within the flipped classroom
Source: BMC Med Educ. 2025 Aug 12;25:1160. doi: 10.1186/s12909-025-07729-z (PMC12341204; doi:10.1186/s12909-025-07729-z)
Supplement: Supplementary file 2 — Supplementary Material 2. [file 12909_2025_7729_MOESM2_ESM.docx]

# Additional file 2

Concepts, measurement items, means, standard deviations (SD), skewness, and kurtosis.

| **Concept** | **Item** | **Mean** | **SD** | **Skewness** | **Kurtosis** |
| --- | --- | --- | --- | --- | --- |
| **Perception of Teachers’ Goals^1^** | My teachers think mistakes are okay as long as we are learning | 5.66 | 1.024 | -1.034 | 2.149 |
|  | My teachers want us to understand our work, not just memorize it | 5.69 | 1.179 | -0.639 | 2.262 |
|  | My teachers really want perceived us to enjoy learning new things | 5.05 | 1.173 | -0.639 | 0.764 |
|  | My teachers recognize us for trying hard | 4.99 | 1.206 | 1.206 | 0.742 |
|  | My teachers give us time to really explore and understand new ideas | 4.44 | 1.346 | 1.346 | -0.142 |
| **Study-effort^1^** | I put a lot of effort into this course | 4.66 | 1.186 | -0.630 | 0.413 |
|  | I tried very hard to do well in this course. | 4.44 | 1.321 | -0.464 | 0.054 |
|  | It was important to me to put effort into this course | 5.71 | .944 | -0.565 | 0.067 |
|  | I actively took part in the discussions and reflection in this course | 4.81 | 1.403 | -0.713 | 0.047 |
| **Perceived Learning Outcomes^2^** | By attending this course, my knowledge regarding situations in relation to cardiac arrest has improved | 3.78 | .682 | -0.342 | 0.546 |
|  | By attending this course, my practical skills in CPR have improved | 3.97 | .669 | -0.367 | 0.736 |
|  | By attending this course, my competence in laws and regulations significant to CPR has improved | 2.80 | .946 | 0.056 | -0.363 |
|  | By attending this course, my competence in critical reflection and assessment of own work has improved | 3.44 | .779 | -0.087 | -0.052 |
|  | By attending this course, my competence of working in a stressful context has improved | 3.31 | .899 | -0.534 | 0.251 |
|  | By attending this course, my competence in communicating precisely in CPR situations has improved | 3.56 | .846 | -0.573 | 0.388 |
|  | By attending this course, my competence in engagement and decision-making in acute and complex situations has improved | 3.52 | .777 | -0.411 | 0.616 |
|  | By attending this course, my cooperation skills in acute and complex situations have improved. | 3.82 | .681 | -0.355 | 0.605 |
|  | By attending this course, my competence in making initiative has improved | 3.73 | .720 | -0.376 | 0.360 |
|  | By attending this course, my ability to perform individually has improved | 3.69 | .739 | -0.330 | 0.185 |
|  | By attending this course, my competence in ethical judgement in connection to CPR has improved | 3.35 | .827 | -0.328 | -0.266 |

^1^Response categories were accompanied by a seven-point scale that ranged from “Absolutely disagree” (1) to “Absolutely agree” (7) with a midpoint of “Neither” (4). ^2^Response categories were accompanied by a five-point scale that ranged from “To a small extent” (1) to “To a large extent” (5). N=351
