# Supplementary material for: Students’ motivation, study-effort and perceptions of teachers’ goals when engaging in a learning design within the flipped classroom
Source: BMC Med Educ. 2025 Aug 12;25:1160. doi: 10.1186/s12909-025-07729-z (PMC12341204; doi:10.1186/s12909-025-07729-z)
Supplement: Supplementary file 3 — Supplementary Material 3. [file 12909_2025_7729_MOESM3_ESM.docx]

# Additional file 1

Measurement instrument: Autonomous motivation when performing different learning activities in the CPR course, with mean (M), standard deviation (SD), skewness, and kurtosis.

| **Concepts** |  | **Item** | **Mean** | **SD** | **Skewness** | **Kurtosis** |
| --- | --- | --- | --- | --- | --- | --- |
| AutonomousMotivation (AM)^1^ | To what extent were the learning activities fun in learning CPR | AM MCQ1 | 2.90 | 1.097 | 0.093 | -0.517 |
|  |  | AM RQI1 | 4.14 | .877 | -0.762 | 0.019 |
|  |  | AM TBL1 | 3.29 | 1.029 | -0.312 | -0.110 |
|  |  | AM Simulation1 | 4.01 | 1.004 | -0.921 | 0.391 |
|  | To what extent were the learning activities useful in learning CPR? | AM MCQ2 | 3.42 | 1.056 | -0.294 | -0.320 |
|  |  | AM RQI2 | 4.49 | .673 | -1.155 | 0.813 |
|  |  | AM TBL2 | 3.46 | 1.057 | -0.453 | -0.175 |
|  |  | AM Simulation2 | 4.30 | .886 | -1.449 | 2.276 |
|  | To what extent were learning activities important for learning CPR? | AM MCQ3 | 3.56 | 1.107 | -0.518 | -0.395 |
|  |  | AM RQI3 | 4.53 | .641 | -1.123 | 0.442 |
|  |  | AM TBL3 | 3.57 | 1.100 | -0.453 | -0.406 |
|  |  | AM Simulation3 | 4.42 | .799 | -1.631 | 2.770 |

^1^Response categories were accompanied by a five-point scale that ranged from “Very little” (1) to “Very much” (5) with a midpoint of “Medium” (3). N=351
